# Structural Neuroplasticity Effects of Singing in Chronic Aphasia

**DOI:** 10.1523/ENEURO.0408-23.2024

**Published:** 2024-05-10

**Authors:** Aleksi J. Sihvonen, Anni Pitkäniemi, Sini-Tuuli Siponkoski, Linda Kuusela, Noelia Martínez-Molina, Sari Laitinen, Essi-Reetta Särkämö, Johanna Pekkola, Susanna Melkas, Gottfried Schlaug, Viljami Sairanen, Teppo Särkämö

**Affiliations:** ^1^Cognitive Brain Research Unit and Centre of Excellence in Music, Mind, Body and Brain, Department of Psychology and Logopedics, Faculty of Medicine, University of Helsinki, Helsinki 00014, Finland; ^2^School of Health and Rehabilitation Sciences, Queensland Aphasia Research Centre and UQ Centre for Clinical Research, The University of Queensland, Brisbane QLD 4072, Australia; ^3^Department of Neurology, University of Helsinki and Helsinki University Hospital, Helsinki 00029, Finland; ^4^HUS Helsinki Medical Imaging Center, Helsinki University Hospital, Helsinki 00029, Finland; ^5^Espoo Hospital, Espoo 00029, Finland; ^6^Private Choir Conductor, Vantaa 01520, Finland; ^7^Department of Neurology, UMass Medical School, Springfield, Massachusetts 01655; ^8^Department of Biomedical Engineering and Institute of Applied Life Sciences, UMass Amherst, Amherst, Massachusetts 01655

**Keywords:** aphasia, music, rehabilitation, singing, stroke, structural connectivity

## Abstract

Singing-based treatments of aphasia can improve language outcomes, but the neural benefits of group-based singing in aphasia are unknown. Here, we set out to determine the structural neuroplasticity changes underpinning group-based singing-induced treatment effects in chronic aphasia. Twenty-eight patients with at least mild nonfluent poststroke aphasia were randomized into two groups that received a 4-month multicomponent singing intervention (singing group) or standard care (control group). High-resolution T1 images and multishell diffusion-weighted MRI data were collected in two time points (baseline/5 months). Structural gray matter (GM) and white matter (WM) neuroplasticity changes were assessed using language network region of interest-based voxel-based morphometry (VBM) and quantitative anisotropy-based connectometry, and their associations to improved language outcomes (Western Aphasia Battery Naming and Repetition) were evaluated. Connectometry analyses showed that the singing group enhanced structural WM connectivity in the left arcuate fasciculus (AF) and corpus callosum as well as in the frontal aslant tract (FAT), superior longitudinal fasciculus, and corticostriatal tract bilaterally compared with the control group. Moreover, in VBM, the singing group showed GM volume increase in the left inferior frontal cortex (Brodmann area 44) compared with the control group. The neuroplasticity effects in the left BA44, AF, and FAT correlated with improved naming abilities after the intervention. These findings suggest that in the poststroke aphasia group, singing can bring about structural neuroplasticity changes in left frontal language areas and in bilateral language pathways, which underpin treatment-induced improvement in speech production.

## Significance Statement

Understanding the neural underpinnings of improved language outcomes in aphasia is vital. We utilize longitudinal neuroplasticity measures of both gray matter (GM) and white matter (WM) and evaluate their contribution to group-based singing treatment effects in chronic aphasia. The results show that singing intervention induced GM and WM neuroplasticity changes in the left frontal language-related structures, but also in the right hemisphere (WM), correlating with improved naming abilities. These results shine light on treatment-induced structural changes in chronic aphasia and improve our understanding of aphasia rehabilitation.

## Introduction

Poststroke aphasia (PSA) is a common and debilitating consequence of stroke, with 60% of the patients remaining aphasic even 1 year after the stroke (Pedersen et al., 2004). PSA incurs enormous socioeconomical burden to the society (Olesen et al., 2012) and is associated with greater extent of rehabilitation services required (Dickey et al., 2010). Due to these profound consequences, effective interventions of aphasia are of great necessity.

The recovery of language functions after PSA is a complex process involving structural reorganization of the spared neurons within the language network according to the neural and neurocomputational models of PSA recovery (Stefaniak et al., 2020). Yet, research mapping treatment-induced structural changes supporting the reorganization processes that bring about beneficial behavioral change in PSA has remained scarce. Small-scale (*N* = 1–8) within-subject longitudinal studies of PSA have reported structural white matter (WM) changes in the left or right arcuate fasciculus (AF) or inferior longitudinal fasciculus following different types of speech therapy, such as constraint-induced language therapy or anomia treatment (Breier et al., 2011; Van Hees et al., 2014; McKinnon et al., 2017) or singing-based therapy, such as melodic intonation therapy (MIT; Schlaug et al., 2009), as well as in the left frontal regions and anterior corpus callosum following excitatory repetitive transcranial magnetic stimulation (Allendorfer et al., 2012). To date, only a single aphasia study has reported structural changes in a randomized controlled trial (RCT), showing that self-managed spoken word comprehension therapy increased gray matter (GM) or WM density in the left and right temporal regions (Fleming et al., 2020). Taken together, our understanding of PSA treatment-induced structural changes that support the recovery of language function is far from comprehensive.

Aphasia can often lead to depression and decreased social functioning, influencing patients’ engagement with the therapy and affecting the outcome (Hilari et al., 2012; Mitchell et al., 2017). Treating associated cognitive impairments and secondary effects of aphasia (depression and social isolation) should also improve communication and, ultimately, quality of life (Doogan et al., 2018). Yet, therapeutic interventions for PSA are implemented traditionally in individual setting, lacking social stimulation. Multifaceted group-based treatments focusing on both language and associated cognitive impairments as well as psychosocial effects of PSA should be of great value and ideal health economically due to limited rehabilitation resources (Doogan et al., 2018). Moreover, as the neuroplasticity changes supporting recovery after stroke can be enhanced by increasing stimulation from the environment (Nithianantharajah and Hannan, 2006; Baroncelli et al., 2010), especially when it involves also a social component (Johansson and Ohlsson, 1996; Venna et al., 2014; Zhang et al., 2021), a combination of increased social, auditory, and cognitive stimulation could provide an avenue to tap into enhanced poststroke neuroplasticity supporting recovery of function in aphasia.

In subacute stroke patients, mere daily vocal music listening (i.e., auditory stimulation) has been shown to improve poststroke language recovery in PSA, increasing the GM volume in the left temporal regions (Sihvonen et al., 2020), strengthening the functional connectivity of the language and default mode networks (Sihvonen et al., 2020, 2021a), and enhancing the structural connectivity of the left frontal aslant tract (FAT) and stimulus-specific activation of its superior frontal termination areas compared with audiobook listening (Sihvonen et al., 2021b). Furthermore, MIT, a singing-based intervention for treating nonfluent aphasia, has been shown to improve connected speech, naming, and repetition (Sparks et al., 1974; Van Der Meulen et al., 2014; Zumbansen et al., 2014) and linking the positive effects to temporal and frontal speech motor areas, either in the left (Belin et al., 1996; Breier et al., 2011) or right (Schlaug et al., 2008; Wan et al., 2014; Tabei et al., 2016) hemisphere. In the healthy older adults, regular singing has recently been linked to enhanced executive function (Pentikäinen et al., 2021; Vetere et al., 2024), frontotemporal auditory functioning (Pentikäinen et al., 2022), structural connectivity (Perron et al., 2021), and structural plasticity in auditory and dorsal speech regions (Perron et al., 2022), suggesting that it may have neuroprotective effects in aging.

The abovementioned increased social, auditory, and stimulation effects could be further strengthened with group-based singing regimens, where, additionally, the spoken language network is stimulated via producing linguistic and musical information via singing (Loui, 2015) that provide further neural stimulation supporting the recovering brain (Murphy and Corbett, 2009). In this vein, a recently published study has explored the behavioral effect of group-based singing intervention in treating patients with chronic aphasia (Siponkoski et al., 2022). Compared to standard care, singing improved responsive speech (Stockert et al., 2020) as indexed by Western Aphasia Battery (WAB) Naming and Repetition indices as well as enhanced long-term communication abilities and reduced family caregiver burden in questionnaires. However, the specific underlying neuroanatomical mediators of recovery of singing-based treatments for PSA are still unclear (Van Der Meulen et al., 2012).

Here, we set out to determine the structural neuroplasticity benefits of group-based singing in an RCT of 28 patients with chronic PSA from Siponkoski et al. (2022). To do so, we evaluated the structural GM and WM neuroplasticity changes after a 4-month singing intervention using language network region of interest (ROI)-based GM analyses and structural connectometry (Yeh et al., 2016a) based on high-resolution T1 images and multishell diffusion-weighted MRI (DW-MRI) data. Previous studies on MIT (Schlaug et al., 2009; Wan et al., 2014) and vocal music interventions (Sihvonen et al., 2021a,b) have shown improved language outcomes in association with neuroplasticity changes in the bilateral frontal areas. Therefore, we hypothesized that the singing intervention induces neuroplasticity changes especially in the left and right frontal areas within the language network that would underpin the improved naming and repetition abilities.

## Materials and Methods

### Subjects and study design

Fifty-four participants with PSA were successfully recruited from the Helsinki region through patient organizations (Helsinki-Uusimaa Stroke Association and Finnish Brain Association) and clinical speech therapists to a registered RCT (ClinicalTrials.gov, NCT03501797). Data collection was performed in two waves, with 33 participants with PSA enrolled and randomized to the trial in Jan. 2018 and 21 participants with PSA in Jan. 2019. Data collection was completed in Dec. 2019. The inclusion criteria were (1) age over 18, (2) Finnish-speaking, (3) time since stroke >6 months, (4) at least mild nonfluent aphasia due to stroke assessed by the Boston Diagnostic Aphasia Examination (BDAE) Aphasia Severity Rating Scale (score ≤4; Goodglass and Kaplan, 1983), (5) normal hearing, (6) no severe cognitive impairment affecting comprehension (e.g., memory disorder or perceptual deficit), (7) no neurological or psychiatric comorbidity or substance abuse, and (8) ability to produce vocal sounds through singing or humming. All participants were interviewed for eligibility by recruiting psychologists (authors A.P. and S-T.S.) and ensured that the patient was able to understand the purpose of the study. The study was conducted in conformance with the Declaration of Helsinki and was approved by the Helsinki University Hospital Ethics Committee. Written informed consent was obtained from all patients and participating caregivers.

**Figure 1.**
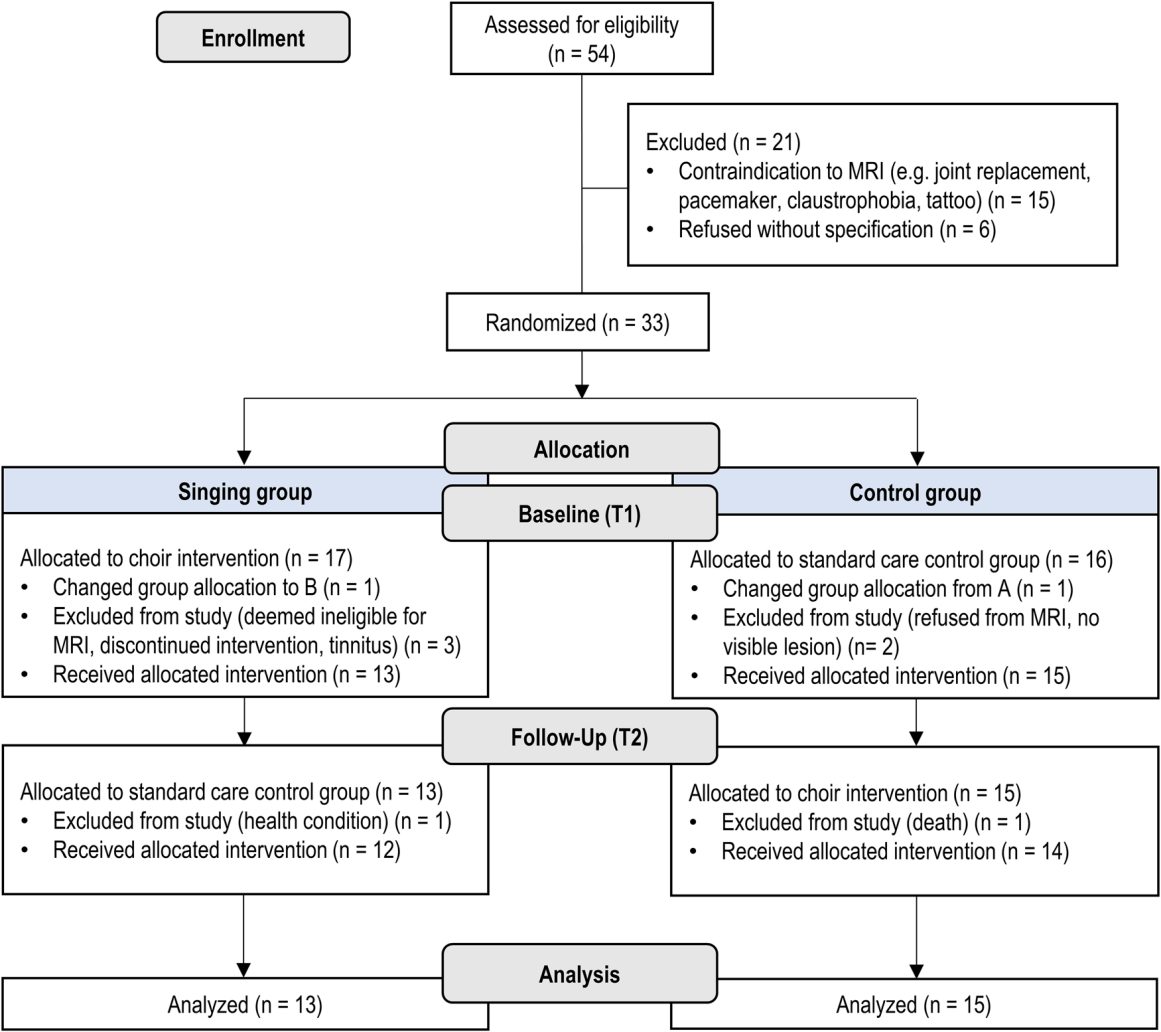
Flowchart.

Of the full sample (*N* = 54), 33 were eligible at the recruitment stage to undergo MRI and were randomly assigned to two groups stratified for aphasia severity (preliminary BDAE score), family caregiver's participation in training sessions, sex, age, and time since stroke (Fig. 1). The randomization was performed using a random number generator by a researcher not involved in data collection. Outcome measurements including neuropsychological assessment and MRI scans were conducted at baseline (T1) and after the intervention period at the 5-month stage (T2). Additionally, the participants filled out a demographical, musical, and clinical background questionnaire at baseline and also reported at T2 the amount of other rehabilitation received between T1 and T2. In total, 28 patients completed the study from T1 to T2 (singing group *N* = 13, control group *N* = 15) and were included in the analyses (Fig. 1, Table 1). All patients received standard chronic stroke care and rehabilitation throughout the study. There were no significant differences between the groups in the amount of received therapy/rehabilitation (Table 1).

**Table 1.** Demographic and clinical characteristics of the patients

	All	Singing group	Control group	*p*-value
	*N* = 28	*N* = 13	*N* = 15	
Demographical				
Age (years)	64.7 (8.4)	64.1 (8.8)	65.2 (8.3)	0.731 (*t*)
Sex (male/female)	14/14	7/6	7/8	0.705 (*χ*^2^)
Handedness (right/left)	23/5	12/1	11/4	0.191 (*χ*^2^)
Education level^a^	3.0 (1.3)	3.2 (1.4)	2.9 (1.3)	0.492 (*U*)
Music background				
Choir singing years	3.3 (9.3)	3.8 (12.4)	2.8 (6.0)	0.389 (*U*)
Singing lessons years	0.6 (3.5)	0.0 (0.0)	1.2 (4.6)	0.352 (*U*)
Instrument lessons years	0.8 (2.3)	0.2 (0.6)	1.3 (3.0)	0.325 (*U*)
Clinical				
Time from injury (months)	78.1 (82.0)	69.7 (77.9)	85.4 (87.3)	0.982 (*U*)
Lesion size (cm^3^)	89.3 (85.3)	119.0 (75.6)	63.5 (87.1)	**0.023 (*U*)**
Type of stroke (ischemic/hemorrhagic)	20/8	11/2	9/6	0.150 (*χ*^2^)
AQ score (mild or moderate/severe)^b^	20/8	8/5	12/3	0.281 (*χ*^2^)
BDAE verbal agility score	7.7 (4.8)	6.5 (5.2)	8.8 (4.2)	0.339 (*U*)
Received rehabilitation (T1–T2)				
Speech therapy^c^	7.3 (10.8)	7.6 (11.6)	6.9 (10.4)	0.717 (*U*)
Physical therapy^c^	6.9 (12.3)	8.5 (15.7)	5.5 (8.8)	1.000 (*U*)
Occupational therapy^c^	2.2 (5.7)	3.9 (8.0)	0.7 (1.5)	0.525 (*U*)
Neuropsychological rehabilitation^c^	0.3 (1.2)	0.0 (0.0)	0.6 (1.6)	0.555 (*U*)

Data are mean (SD) unless otherwise reported. Bold values denote statistical significance at *p* < 0.05. *t*, *t* test; *χ*^2^, chi-square test; *U*, Mann–Whitney *U* test; AQ, aphasia quotient; BDAE, Boston Diagnostic Aphasia Examination.

a Education level according to the UNESCO International Standard Classification of Education: range 1 (primary education) to 6 (doctoral or equivalent level).

b Aphasia severity based on the AQ score: 0–50 = severe, 51–100 = mild/moderate.

c Data are mean (SD) in hours between T1 and T2.

### Intervention

The duration of the intervention was 16 weeks, consisting of group training (once a week, 90 min/session, total 24 h) and self-training at home with a tablet computer software (target: three sessions/week, 30 min/session, total 24 h). Each group training session comprised group-based singing (60 min) and adapted group-level MIT (30 min) [see (Siponkoski et al., 2022) for details]. The sessions were arranged at the Aphasia Centre of the Helsinki-Uusimaa Stroke Association and were implemented by a music therapist and choir conductor team. Each patient had the opportunity to invite one caregiver or family member to participate in the sessions with them. Group-based singing included breathing and vocal exercises, vocal improvisation, and group singing with choral elements of 10 songs that were selected and arranged to be suitable for the patients. Group-level MIT comprised production of formulaic phrases utilizing the elements of MIT: intoning the phrases with simple melodic structure, tapping the rhythm with the left hand, and progressing hierarchically from unison production to repetition and from singing to spoken prosody (Schlaug et al., 2009). Home training sessions included self-training with a tablet computer, a headset, and an application developed together with Outloud, a Finnish software company. The application included all songs rehearsed in the training sessions and enabled progressive training with two different auditory models (vocal and instrumental melody) and two different visual aids (visual-kinetic model and visual-text model, that is, seeing the mouth movements of the singer and lyrics on the screen) that could be selected separately or in any combination. The software analyzed key acoustic features of the voice to provide immediate feedback of the singing performance. The amount of home training was tracked with the app log files and was 10 h 24 min on average for the singing group (SD 6 h 51 min).

### Language assessment

The language assessment was conducted by trained psychologists for each patient at all time points, blinded to the group allocation of the participants. At baseline, WAB Aphasia Quotient (Kertesz, 2007), indicating the overall severity level of the aphasia, was calculated from the Spontaneous speech, Repetition, Naming, and Comprehension (estimated based on the Sequential commands subtest) indices. Here, based on the primary spoken language production outcome measure used in the study (Siponkoski et al., 2022), we focused a priori on naming and repetition (as indicated by WAB Naming and Repetition indices; Kertesz, 1982) as the primary aspects of test-assessed language functioning improved by singing intervention due to their strong neurobiological foundation (Geva et al., 2012; Hickok, 2012; Alyahya et al., 2020; Døli et al., 2021), providing commonly used and quantifiable measures of language function that can serve as feasible counterparts of structural brain changes in PSA, thereby also reducing dimensionality in analysis. Additionally, to control for possible group differences at baseline, we evaluated motor speech production (apraxia of speech) using the articulatory agility subtest of BDAE (Goodglass and Kaplan, 1983).

### MRI data acquisition and preprocessing

Patients were scanned on a 3T Siemens Skyra scanner at the Department of Radiology of the Helsinki University Hospital. For each patient, high-resolution T1-weighted anatomical images (TR = 1,800 ms; TE = 2.27 ms; TI = 900 ms; field of view = 250 × 250 mm; voxel size = 1 × 0.98 × 0.98 mm^3^) and multishell DW-MRI scan (TR = 5,000 ms, TE = 104 ms, field of view = 240 × 240 mm, voxel size = 2 × 2 × 2 mm^3^, directions = 142, *b*-max = 2,500 s/mm^2^) with 13 nondiffusion-weighted volume and 130 diffusion-weighted volumes (30 volumes with *b* = 1,000 s/mm^2^ and 100 volumes with *b* = 2,500 s/mm^2^) were acquired.

**Figure 2.**
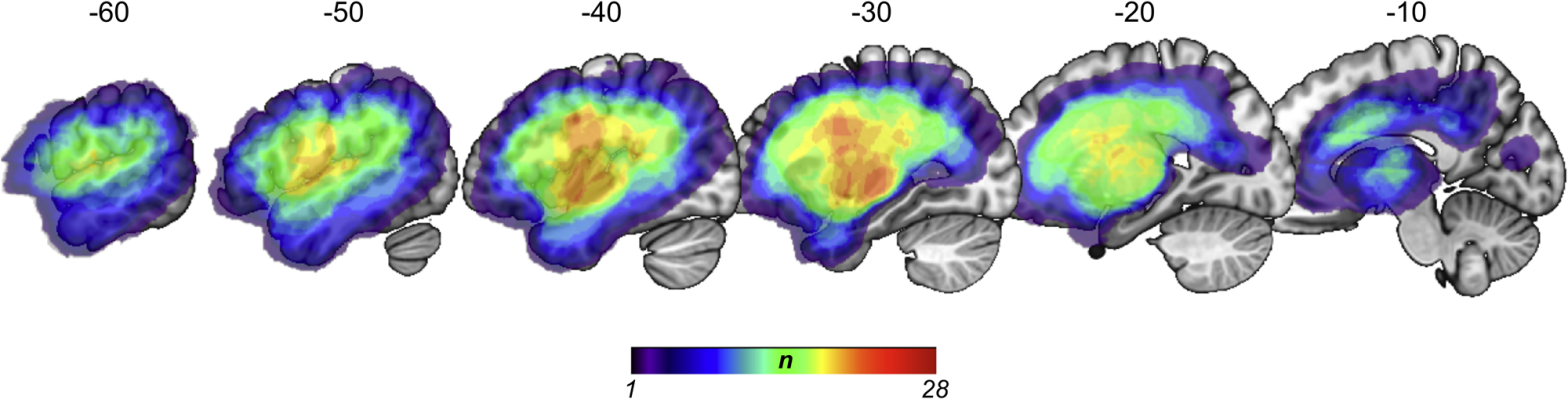
Lesion overlap map of all patients (*N* = 28). The *n*-value represents the number of patients with a lesion to a specific voxel.

MRI data were preprocessed using the Statistical Parametric Mapping software (SPM12, Wellcome Department of Cognitive Neurology, UCL, www.fil.ion.ucl.ac.uk/spm/) under MATLAB 9.4.0. To achieve optimal normalization of MRI images containing stroke lesions, cost function masking (CFM) was applied (Brett et al., 2001). This exact approach has been widely used in stroke patients (Crinion et al., 2007; Ripollés et al., 2012; Sihvonen et al., 2020) and prevents postregistration lesion shrinkage and out-of-brain distortions. The CFMs were defined separately in each time point by creating precise binary masks of the lesions by manually delineating them to the individual T1 images slice-by-slice by authors A.J.S. and N.M-M. using the MRIcron software package (http://people.cas.sc.edu/rorden/mricron/index.htm). Lesion masks were verified by a neuroradiologist (Fig. 2). T1 images and lesion masks were then reoriented according to the anterior commissure and segmented using unified segmentation (Ashburner and Friston, 2005) with medium regularization and SPM12 IXI tissue probability maps. Individual lesion maps were used to apply CFM during the preprocessing. Due to large lesion sizes, damaged voxels were masked out to achieve accurate segmentation and spatial normalization. The segmented GM probability maps were then modulated, resampled to 2 × 2 × 2 mm^3^ voxel size, and normalized to Montreal Neurological Institution (MNI) space, together with the binarized lesion maps. Residual interindividual variability was reduced by smoothing the GM probability maps using an isotropic spatial filter (FWHM = 6 mm). Lastly, the segmented GM probability maps were visually inspected for segmentation errors and distortions to ensure optimal segmentation.

### DW-MRI data preprocessing and reconstruction

First, the DW-MRI data were denoised for thermal noise with the MP-PCA method (Veraart et al., 2016) using a denoise tool from MRTrix3 (https://www.mrtrix.org/; Tournier et al., 2019) and corrected for Gibbs ringing based on local subvoxel shifts (Kellner et al., 2016). The b-table was checked by an automatic quality control routine to ensure its accuracy (Schilling et al., 2019). The DW-MRI data were reconstructed in the MNI space using DSI Studio (http://dsi-studio.labsolver.org, version April 7, 2021) and q-space diffeomorphic reconstruction (Yeh and Tseng, 2011) that allows the construction of spin distribution functions (Yeh et al., 2010). Normalization to the MNI space provides a direct way to analyze, for example, group differences. Normalization was carried out using the anisotropy map of each participant, and a diffusion sampling length ratio of 1.25 was used. The data output was resampled to 2 mm isotropic resolution. The quality of the normalization was inspected using the *R*^2^ values denoting goodness of fit (*R*^2 ^> 0.6) between the participant's anisotropy map and template. Furthermore, each participant's forceps major and minor were inspected and used as an anatomical benchmark to confirm the normalization quality (Hula et al., 2020). The restricted diffusion was quantified using restricted diffusion imaging (Yeh et al., 2017) and quantitative anisotropy (QA) was extracted as the local connectome fingerprint (Yeh et al., 2016b) and used in the connectometry analysis. QA has been shown to outperform traditional fractional anisotropy (FA) by being more specific to individual's connectivity patterns (Yeh et al., 2016b) and less susceptible to the partial volume effect of crossing fibers and free water as well as to provide better resolution in tractography (Yeh et al., 2013).

### Regions of interest

As we expected the intervention to induce treatment-related and activity-dependent neuroplasticity effects in brain regions activated by the singing intervention (Murphy and Corbett, 2009), we focused the analyses on neural structures related to both singing and aphasia recovery. To do this, a probabilistic human brain atlas, Brainnetome atlas (https://atlas.brainnetome.org/; Fan et al., 2016), explicitly accommodating intersubject variability in anatomy, was used to define the ROIs. Four regions from each hemisphere were derived from the Brainnetome atlas: Brodmann area (BA) 44, BA45, ventral premotor cortex (vPMC), and posterior middle temporal gyrus (pMTG). All of these areas have been implicated in singing [BA44/45 (Kleber et al., 2013; Zarate, 2013; Marchina et al., 2023), vPMC (Callan et al., 2006; Kleber et al., 2013; Marchina et al., 2023), pMTG (Whitehead and Armony, 2018; Marchina et al., 2023)] or in supporting recovery in PSA [BA44/45 (Crinion and Price, 2005; Turkeltaub et al., 2011; Hartwigsen and Saur, 2019; Stefaniak et al., 2021), vPMC (Saur et al., 2006; Seghier et al., 2014a), pMTG (Crinion and Price, 2005; Griffis et al., 2017)]. BA44 and BA45 (together known as Broca's area) were investigated separately given their established differentiation in function (Gough et al., 2005) and connectivity (Anwander et al., 2007).

### Connectometry analysis

Connectometry (Yeh et al., 2016a) analyses were carried out using DSI Studio (http://dsi-studio.labsolver.org, version April 7, 2021). Connectometry is a reasonably new statistical method that includes mapping and analysis of local connectomes, that is, the degree of connectivity between adjacent voxels within a WM tract defined by the density of the diffusing spins. As a result, connectometry identifies the segments of WM fiber bundles that exhibit significant association with the study variable, here group over time. Unlike traditional FA-based structural connectome analyses, which identify differences in the mean values for the whole WM tract or using voxel-based FA values, connectometry uses QA, a measure based on the diffusion orientation distribution function (ODF), to track only the segment of the fiber bundle that exhibits significant association with the study variable or group difference. To do this, DW-MRI data are reconstructed into a standard template space (MNI) onto a local connectome matrix from the studied sample. Study-relevant variables or group information are then associated with this local connectome matrix to identify local connectomes expressing significant associations with the variable of interest. Using diffusion ODF-based measure (QA) for resolving multiple fibers, these local connectomes are then tracked along the core pathway of a fiber bundle using a fiber tracking algorithm within the Human Connectome Project tractography atlas (HCP-1065) based on 1,065 subjects and compared with a null distribution of coherent associations using permutation statistics. In summary, connectometry analyzes significant QA associations with a variable of interest or QA differences between two groups along the pathways themselves as compared with mean FA in a voxel or representing a whole tract. The analysis then outputs the significant segments of the connectome and tracts that were significantly associated between the group difference and the study variable. As the DW-MRI data are reconstructed into standard space and tracking is based on template, it also minimizes bias and variability induced by manual tracking in which, for example, slightly increasing the size of ROIs used to dissect tracts drastically changes the resulting number of streamlines and the volume they occupy, inducing significant variability within protocols and across subjects (Rheault et al., 2020; Schilling et al., 2021). The minimum length is set by voxel threshold (here 20 voxels).

A statistical model utilizing nonparametric Spearman rank-based correlation was built to consider the nonlinear effect of the group (singing group vs control group, control group vs singing group) and the longitudinal change of QA. In other words, the model compared the longitudinal (T2 > T1) significant QA changes across the structural connectome between the groups to evaluate possible treatment-related neuroplasticity changes and whether they were larger in the singing group or in the control group. Due to the group difference observed in lesion size, it was included as a nuisance variable in the analyses (Table 1). The eight selected ROIs were used as seeding regions. Local connectomes with T-score exceeding 2 were selected (Hula et al., 2020) and tracked using a deterministic fiber tracking algorithm (Yeh et al., 2013) to obtain correlational tractography. The tracks were filtered by topology-informed pruning (Yeh et al., 2019) with four iterations, and a length threshold of 20-voxel distance was used to identify significant tracts. Bootstrap resampling with 10,000 randomized permutations was used to obtain the null distribution of the track length and estimate the false discovery rates (FDRs). The alpha level was set to *p*_FDR _< 0.05.

### GM neuroplasticity analysis

The GM volume in each of the eight ROIs in two time points (T1 and T2) was extracted for all participants using SPM12 and exported to SPSS (IBM SPSS Statistics for Windows, v.27.0.: IBM, https://www.ibm.com/products/spss-statistics). A multivariate ANOVA across groups (the independent variable), including age, sex, education, total intracranial volume, and lesion size as nuisance variables (Barnes et al., 2010; Table 1), was calculated for the GM volume change (the dependent variable) in T2 > T1 (treatment-related neuroplasticity changes vs control). The total intracranial volume and lesion size did not correlate significantly (*r* = 0.163; *p* = 0.407). FDR correction was applied to control for multiple comparisons (*N* = 8), and the alpha level was set to *p*_FDR _< 0.05.

Additional voxel-based morphometry (VBM) analysis was performed using SPM12 (Wellcome Department of Cognitive Neurology, UCL, www.fil.ion.ucl.ac.uk/spm/) under MATLAB 9.4.0 to evaluate the voxel-wise GM changes in association with the intervention. To assess the longitudinal differences in GM volume changes over time between the singing group and the control group, GM difference images were first calculated with ImCalc by subtracting each patient's GM probability map at baseline from the 5-month follow-up GM probability map. Then, the longitudinal individual preprocessed GM images were submitted to second-level independent sample *t* test analyses with group (singing group and control group) as factor and age, sex, education, time from stroke, total intracranial volume, and lesion size as nuisance variables. Three different *t* tests were calculated: (1) an unrestricted whole-brain analysis, (2) a voxel-wise analysis focused within the language network based on meta-analysis (https://neurosynth.org/analyses/terms/language/), and (3) a voxel-wise analysis focused within the study-specific ROIs. All results were thresholded at an uncorrected *p* < 0.005 threshold at the voxel level, and standard SPM family-wise error rate (FWE) cluster-level correction based on random field theory with a *p*_FWE _< 0.05 was used. Only clusters surviving FWE-corrected *p* < 0.05 at the cluster level are reported.

### Data availability

Anonymized data reported in this manuscript are available from the corresponding author upon reasonable request and subject to approval by the appropriate regulatory committees and officials. We have reported how we determined our sample size, all data exclusions (if any), all inclusion/exclusion criteria, whether inclusion/exclusion criteria were established prior to data analysis, all manipulations, and all measures in the study.

## Results

First, by using two separate univariate ANOVAs, we confirmed that the singing and control groups did not differ in baseline WAB Naming (*p*_FDR _= 0.585) and WAB Repetition (*p*_FDR _= 0.585) scores (Table 2). Then, using two separate univariate ANOVAs, we evaluated whether the singing intervention was associated with language function improvements in the current sample patients with chronic PSA from the original trial (Siponkoski et al., 2022). Due to the group difference (Table 1), the analysis was adjusted for lesion size. The ANOVAs showed that the singing group significantly improved in WAB Naming [*F*_(1,25)_ = 10.98; *p*_FDR _= 0.006; *η*_p_^2^ = 0.305] but not in WAB Repetition [*F*_(1,25)_ = 1.24; *p*_FDR _= 0.277; *η*_p_^2^ = 0.047] compared with the control group between T1 and T2 (ΔT2–T1).

**Table 2.** Spoken language and neuroplasticity analysis outcomes

Measure	Group	T1 mean (SD)	T2 mean (SD)	ΔT2 > T1 mean (SD)	Baseline diff. (*p*_FDR_-value)	ΔT2 > T1 (*p*_FDR_-value)
Spoken language outcomes						
WAB Naming	Singing	5.0 (3.6)	5.7 (3.8)	0.7 (0.7)	0.585	**0.006**
	Control	7.4 (3.6)	7.4 (3.5)	0.0 (0.5)		
WAB Repetition	Singing	5.4 (3.6)	5.7 (3.4)	0.3 (0.7)	0.585	0.277
	Control	7.6 (3.4)	7.6 (3.3)	0.0 (0.4)		
Neuroplasticity outcomes						
GMV left BA44^a^	Singing	747 (568)	807 (586)	61 (108)	0.945	**0.008**
	Control	854 (387)	713 (427)	−140 (141)		
GMV right BA44^a^	Singing	1,619 (159)	1,628 (278)	9 (99)	0.718	0.587
	Control	1,798 (336)	1,743 (324)	−55 (149)		
GMV left BA45^a^	Singing	387 (304)	414 (306)	28 (43)	0.945	**0.024**
	Control	409 (240)	336 (263)	−73 (78)		
GMV right BA45^a^	Singing	873 (159)	891 (173)	19 (61)	0.720	0.759
	Control	924 (201)	913 (164)	−11 (110)		
GMV left vPMC^a^	Singing	884 (567)	941 (607)	57 (203)	0.718	**0.024**
	Control	1,106 (400)	913 (460)	−195 (151)		
GMV right vPMC^a^	Singing	1,783 (293)	1,775 (309)	−8 (105)	0.833	0.876
	Control	1,827 (448)	1,810 (370)	−21 (172)		
GMV left pMTG^a^	Singing	1,103 (718)	1,090 (724)	−13 (76)	0.718	0.268
	Control	1,383 (430)	1,431 (591)	47 (56)		
GMV right pMTG^a^	Singing	2,205 (533)	2,242 (551)	37 (137)	0.718	0.777
	Control	2,498 (503)	2,513 (477)	14 (74)		
WM left AF^b^	Singing	0.10 (0.04)	0.11 (0.05)	0.01 (0.01)	0.480	**0.005**
	Control	0.11 (0.04)	0.11 (0.05)	0.00 (0.02)		
WM left FAT^b^	Singing	0.13 (0.03)	0.14 (0.04)	0.01 (0.02)	0.523	**0.004**
	Control	0.14 (0.05)	0.13 (0.06)	−0.01 (0.02)		
WM right FAT^b^	Singing	0.19 (0.03)	0.20 (0.03)	0.01 (0.01)	0.815	**0.004**
	Control	0.20 (0.03)	0.19 (0.02)	0.00 (0.02)		
WM left SLF^b^	Singing	0.10 (0.05)	0.11 (0.05)	0.01 (0.01)	0.480	**0.005**
	Control	0.11 (0.04)	0.11 (0.05)	0.00 (0.02)		
WM right SLF^b^	Singing	0.21 (0.03)	0.22 (0.03)	0.01 (0.01)	0.480	**0.004**
	Control	0.20 (0.03)	0.20 (0.03)	0.00 (0.01)		
WM left CS^b^	Singing	0.09 (0.04)	0.10 (0.04)	0.01 (0.01)	0.480	**0.004**
	Control	0.10 (0.04)	0.10 (0.05)	0.00 (0.01)		
WM right CS^b^	Singing	0.16 (0.04)	0.17 (0.04)	0.01 (0.01)	0.555	**0.004**
	Control	0.17 (0.03)	0.17 (0.02)	0.00 (0.01)		
WM CC^b^	Singing	0.14 (0.03)	0.15 (0.03)	0.01 (0.01)	0.480	**0.005**
	Control	0.15 (0.03)	0.15 (0.03)	−0.01 (0.01)		

Data are mean (SD) unless otherwise reported. Bold values denote statistical significance at *p* < 0.05. *P*-values are FDR corrected.

a Volume in mm^3^.

b Normalized QA.

### Intervention-induced WM neuroplasticity changes

First, using a nonparametric Spearman correlational model, we confirmed that the singing and control groups did not differ in baseline QA in any WM tract (*p*_FDR _= 0.140–1.000).

**Figure 3.**
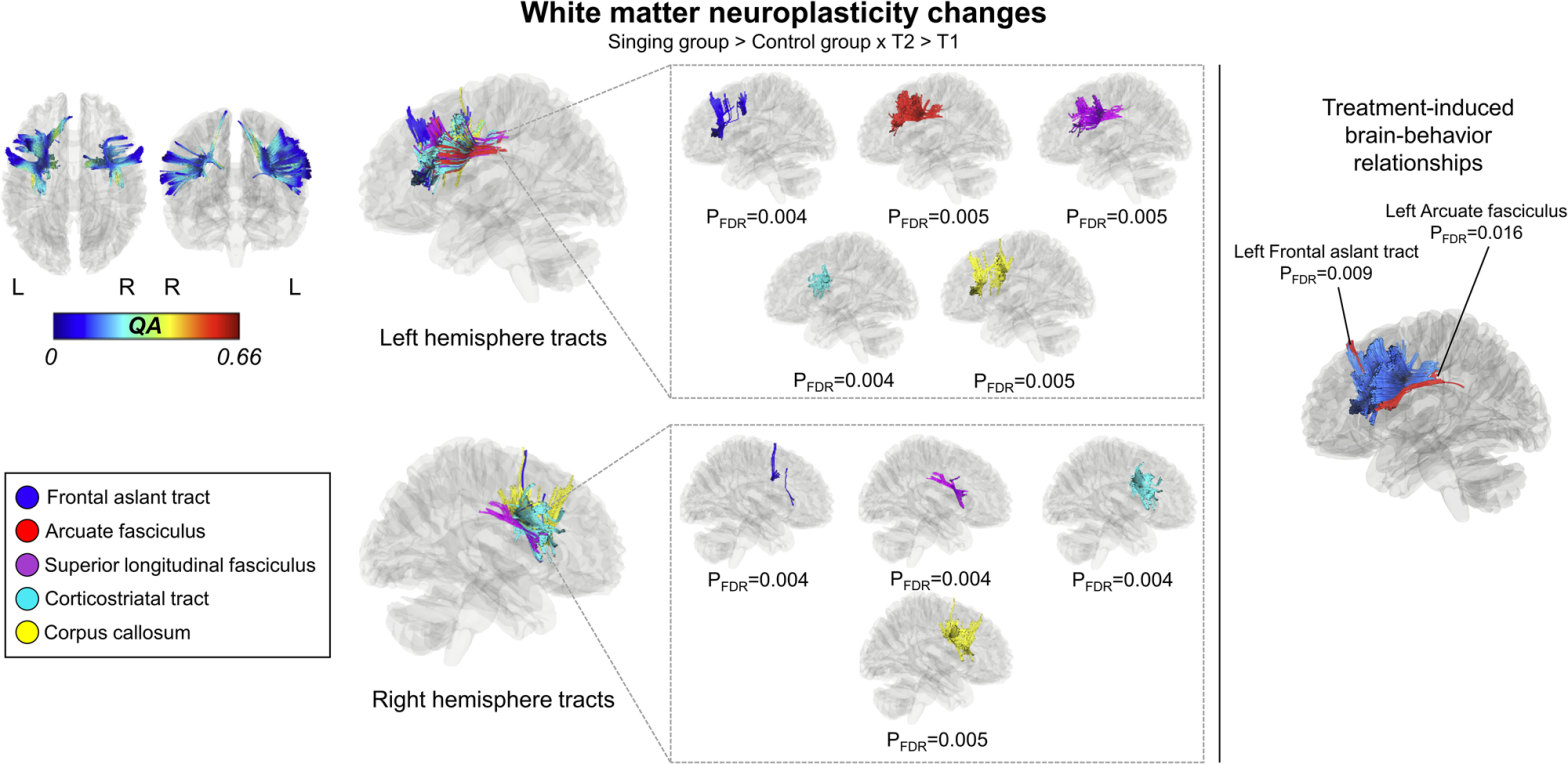
Treatment-induced WM neuroplasticity changes. Connectometry results displaying the significant segments of the tracts with longitudinal QA increases significantly associated with singing group versus control group between T1 and T2 (ΔT2–T1; left) and longitudinal QA change correlation with improved naming (right). FDR, false discovery rate; L, left; QA, quantitative anisotropy; R, right.

Compared to the control group, the singing group showed greater increase in QA in the left AF (*p*_FDR _= 0.005), FAT (*p*_FDR _= 0.004), superior longitudinal fasciculus (SLF: *p*_FDR _= 0.005), and corticostriatal tract (*p*_FDR _= 0.004), and in the right FAT (*p*_FDR _= 0.004), SLF (*p*_FDR _= 0.004) and corticostriatal tract (*p*_FDR _= 0.004) as well as in the the corpus callosum (*p*_FDR _= 0.005) in ΔT2–T1 (Fig. 3).

### Intervention-induced GM neuroplasticity changes

First, using separate univariate ANOVAs, we confirmed that the singing and control groups did not differ in baseline GM volume in any of the chosen ROIs (*p*_FDR _= 0.822–0.867).

**Figure 4.**
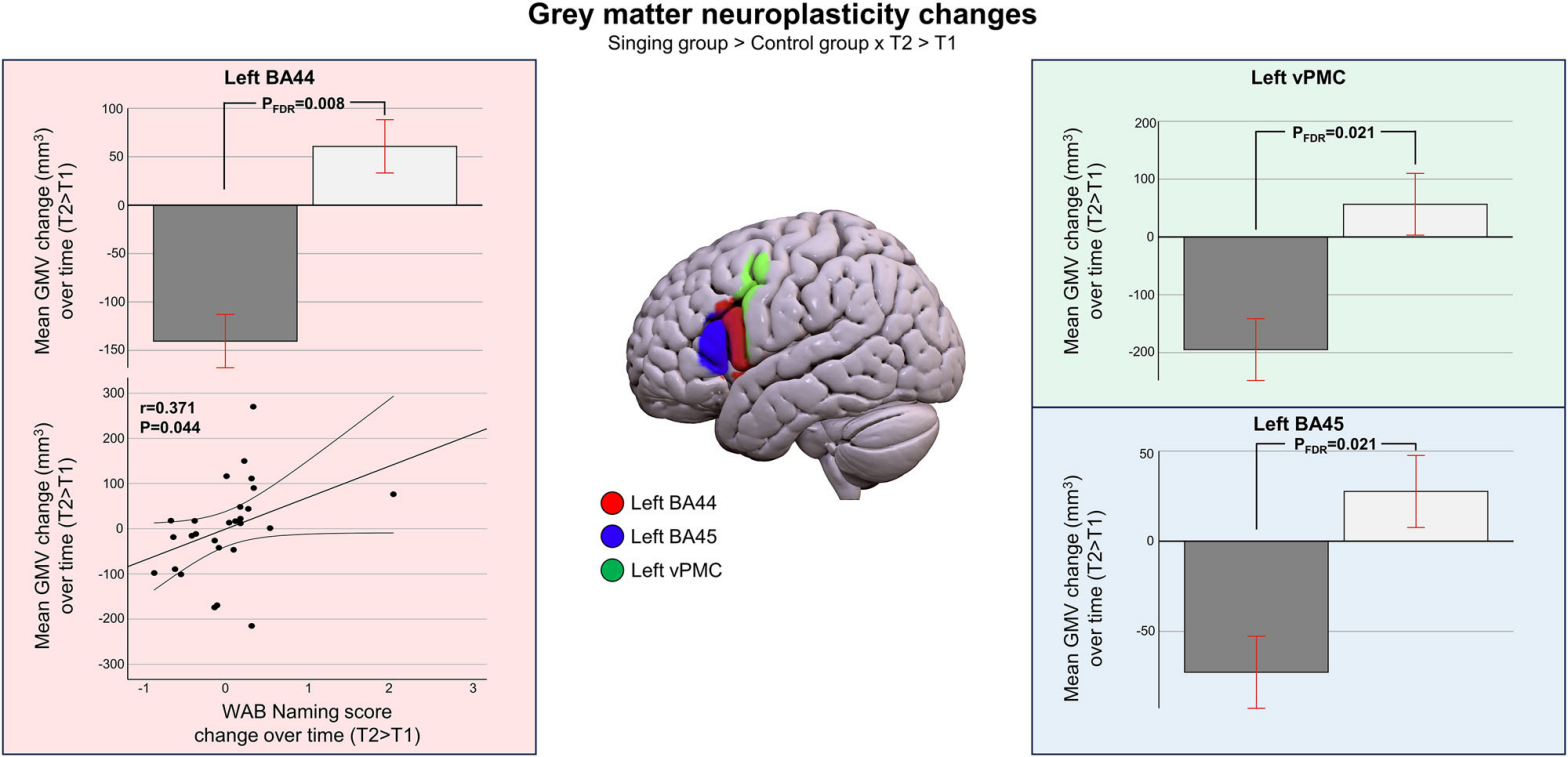
Treatment-induced GM neuroplasticity changes. Longitudinal GM volume increases (singing group > control group) in T2 > T1 and longitudinal GM volume change correlation with improved naming. Additional exploratory voxel-wise analyses are reported in Extended Data Figure 4-1. Bar plots for mean group GM volume changes are shown: bar, mean; error bar, standard error of mean. BA, Brodmann area; FDR, false discovery rate; vPMC, ventral premotor cortex.

In the multivariate ANOVA evaluating treatment-related GM changes (ΔT2–T1), there was a statistically significant difference in the longitudinal change in the GM volume between the groups [*F*_(8,19)_ = 3.759; *p* = 0.020; Wilk's *Λ *= 0.285; *η*_p_^2^ = 0.715]. Therefore, eight separate univariate ANOVAs (i.e., one for each ROI) were performed. These revealed that the GM volume change (ΔT2–T1) of the left BA44 [*F*_(1,19)_ = 20.785, *p*_FDR _= 0.008; *η*_p_^2^ = 0.522; singing group_mean _= 61 mm^3^, control group_mean _= −140 mm^3^], left BA45 [*F*_(1,19)_ = 9.772, *p*_FDR _= 0.024; *η*_p_^2^ = 0.340; singing group_mean _= 28 mm^3^, control group_mean _= −73 mm^3^], and left vPMC[*F*_(1,19)_ = 8.607, *p*_FDR _= 0.024; *η*_p_^2^ = 0.312; singing group_mean _= 57 mm^3^, control group_mean _= −195 mm^3^] showed greater increase in the singing group than in the control group (A > B; Fig. 4). ANOVAs for the other ROIs were not significant after FDR correction.

10.1523/ENEURO.0408-23.2024.f4-1 Figure 4-1 Voxel-wise analysis of the treatment-induced grey matter neuroplasticity changes. Longitudinal GM volume increases (Singing group>Control Group) in T2>T1 from (left) the whole-brain voxel-wise analysis, (middle) the voxel-wise analysis within the language network derived from meta-analysis (https://neurosynth.org/analyses/terms/language/) and (right) the voxel-wise analysis within the study-specific regions of interest. BA=Brodmann area, FWE=Family-wise error rate, vPMC=ventral premotor cortex. Download Figure 4-1, TIF file.

In addition to the a priori defined ROI-based analysis, longitudinal treatment-related GM volume changes were also assessed using three different voxel-wise analyses: (1) an unrestricted whole-brain analysis, (2) a voxel-wise analysis focused within the language network based on meta-analysis (https://neurosynth.org/analyses/terms/language/), and (3) a voxel-wise analysis focused within the study-specific ROIs. Similar to the ROI-based analysis, age, sex, education, total intracranial volume, and lesion size were added as nuisance variables. In all three longitudinal VBM analyses, the GM volume increased more in the singing group than in the control group in one cluster comprising the left BA44 and vPMC (peak MNI coordinate = −56, 16, 18; Extended Data Figure 4-1). Both within language network (cluster size = 512 voxels, *T*-value = 6.43, *p*_FWE _= 0.038) and within study-specific ROIs (cluster size = 167 voxels, *T*-value = 6.45, *p*_FWE _= 0.041) analyses were statistically significant, but the GM volume change failed to reach statistical significance in the unrestricted whole-brain analysis (cluster size = 366 voxels; *T*-value = 5.96; *p*_FWE _= 0.263).

### Brain–behavior relationships

To investigate treatment-induced brain–behavior relationships, we first evaluated the longitudinal QA change associations with improved naming, as the functional restoration in PSA mostly relies upon the structural remodeling of the injured networks (Stefaniak et al., 2020). Therefore, a nonparametric Spearman correlational model (T2 > T1) was built to derive the correlational tractography within the significant WM findings evaluating the longitudinal change of QA correlated with the improvement in WAB Naming. In ΔT2 > T1, increased QA in the left FAT (*p*_FDR _= 0.009) and AF (*p*_FDR _= 0.016) correlated with improved naming (Fig. 3).

Evaluation of the relationship between improved naming scores and GM volume changes were restricted to the left BA44 to which both left AF and FAT are frontally terminated (Thiebaut de Schotten et al., 2012). Partial correlation (Pearson’s, one-tailed, controlling for the same nuisance variables as in the initial GM volume analysis, i.e., age, sex, education, total intracranial volume, and lesion size) using SPSS showed that increased GM volume (ΔT2–T1) in the left BA44 correlated with improved naming abilities (*r* = 0.371; *p* = 0.044; Fig. 4).

## Discussion

This study set out to determine the structural GM and WM benefits of group-based singing in PSA rehabilitation and their correlation to improved language outcome. Our novel main findings were that group-based singing enhanced structural WM connectivity in both the left and right hemispheres within the language network and the GM volume in the left language-related frontal areas compared with the control group. The left frontal neuroplasticity effects correlated with improved naming abilities. The present study provides the first evidence on the neural benefits of group-based singing that supports language recovery in PSA and extends previous results on the effects of music-based interventions in stroke rehabilitation, including MIT (Schlaug et al., 2009; Marchina et al., 2023) and music listening (Sihvonen et al., 2017a, 2020, 2021b). These results are important in (1) providing evidence of treatment-induced structural changes in chronic PSA, (2) improving our understanding of chronic PSA rehabilitation, and (3) determining targets and mediators of music-based rehabilitation strategies (Sihvonen et al., 2017a).

In aphasia, recovery relies on the ability of the spared neurons to remodel the injured network (Kiran et al., 2019). The recovery processes in PSA exploit activity-dependent neuroplasticity mechanisms (Murphy and Corbett, 2009) within the language network (Stefaniak et al., 2020, 2021), that is, increased stimulation through iterative utilization of language processes, and therefore activating the language network supports the recovering brain by increasing, for example, dendritic spine density and neurotrophic factor levels (Carmichael et al., 2001; Nithianantharajah and Hannan, 2006). In the context of neural stimulation, music-based interventions, such as singing, are feasible tools to promote language network recovery in PSA (Särkämö and Sihvonen, 2018). First, simply listening to vocal music has been demonstrated to activate language-related regions of the brain, even in the case of acute stroke (Sihvonen et al., 2017b). Moreover, daily listening to vocal music poststroke has been linked to improved language recovery in PSA, evidenced by improved language skills and verbal memory and increased GM volume within the left temporal regions (Sihvonen et al., 2020). The vocal music listening intervention also enhances the functional connectivity within the language and default mode networks (Sihvonen et al., 2020, 2021a), and strengthens the structural connectivity of the left FAT and stimulus-specific activation of its superior frontal termination areas compared with audiobook listening (Sihvonen et al., 2021b). Compared to mere vocal music listening, choir singing should be superior in administering neural stimulation and providing more fertile environment for recovery as it incorporates multiple elements such as the production of words through singing, physiological effects of singing, the experience of singing with others, the perception of sung music, social interaction, and the learning of new songs and lyrics. In theory, these combined factors create a more conducive environment for neural stimulation and recovery (Murphy and Corbett, 2009).

Second, singing and speech share core neuronal circuitry within the left hemisphere (Pitkäniemi et al., 2023). Singing also binds linguistic and musical information into a unified representation and naturally increases connectedness between syllables and words and, in this respect, resembles connected spoken language production. Compared to speaking, singing engages bilateral language-related frontotemporal areas more extensively (Callan et al., 2006; Schön et al., 2010) and requires multiple neural circuits to operate in concert (Loui, 2015). The classical notion in neurology is that even patients with severe PSA can retain the ability to sing lyrics of familiar songs (Johnson and Graziano, 2015). Sung information is also accessible to patients with PSA who have been shown to repeat and recall more words when singing than when speaking (Racette et al., 2006; Leo et al., 2018). This evidence suggests that singing can provide an avenue for language rehabilitation in PSA. Indeed, singing-based interventions such as MIT have been shown to improve language recovery in nonfluent PSA (Sparks et al., 1974; Van Der Meulen et al., 2014; Zumbansen et al., 2014) with associated positive neuroplasticity effects reported in language-related frontotemporal areas bilaterally (Belin et al., 1996; Schlaug et al., 2008; Breier et al., 2011; Wan et al., 2014; Tabei et al., 2016).

In accordance with the abovementioned, our current findings revealed that group-based singing enhanced WM connectivity in both hemispheres, but with left-hemispheric dominance. Treatment-related changes correlating with improved naming abilities comprised the left AF and FAT, damage of which has been associated with speech production outcome in PSA (Fridriksson et al., 2013; Alyahya et al., 2020). Changes were also observed in the corpus callosum, SLF, and corticostriatal tract bilaterally. These observations might lend to two distinct mechanisms, that is, treatment-related changes within the language network and in the shared structures between the language and domain-general networks. According to the neurocomputational model of PSA recovery, initially damaged AF/SLF undergoes plasticity-related changes during the recovery (Stefaniak et al., 2020). The corpus callosum has been shown to play a critical role in language comprehension in integrating prosodic and syntactic information (Sammler et al., 2010), and its treatment-related changes after singing-based treatments, combining melody, rhythm, and linguistic information, are reasonable findings. In contrast, treatment-related plasticity changes in the corticostriatal tracts might reflect more domain-general network effects as corticostriatal systems have been shown to play a domain-general regulatory role in language operations (Copland et al., 2021). Moreover, the proposed neuroanatomical model supporting singing center on the left AF/SLF but also includes the left FAT as well as ventral tracts (Loui, 2015). The present findings conform with this model and the previous neuroanatomical evidence on the core neuronal circuitry underpinning singing of words in aphasia (Pitkäniemi et al., 2023) as well as with the previous small-scale PSA treatment-induced WM findings (Schlaug et al., 2009; Breier et al., 2011; Allendorfer et al., 2012; Van Hees et al., 2014).

Group-based singing-induced GM plasticity changes that correlated with improved naming abilities were observed in the left BA44 where the left AF/SLF and FAT cortically terminate (Thiebaut de Schotten et al., 2012). The intervention group showed longitudinally slightly increased GM volume in the left BA44, whereas the control group showed a decline in that area. This most likely owes to the accelerated brain atrophy rate after stroke, which is 2–4 times greater than in healthy controls (Brodtmann et al., 2020, 2021; Salah Khlif et al., 2022). Lesioned areas have been shown to lead to further neuronal decay, even in the chronic poststroke stage, with a median rate of 1,590 mm^3^ per year (Seghier et al., 2014b). In comparison, the mean left BA44 GM volume decrease in the control group in the present study was 140 mm^3^ in 5 months. Similarly, WM neurodegeneration in ipsi- and contralesional tracts continues to be greater in stroke survivors compared with the healthy population (Egorova-Brumley et al., 2023). In a recent study on patients with PSA, most patients showed evidence of lesion expansion and that it was associated with further declining language performance (Johnson et al., 2023). The poststroke brain atrophy rates may serve as biomarkers reflecting treatment response for interventions to reduce poststroke secondary degeneration and vascular cognitive impairment (Brodtmann et al., 2021). For these reasons, interventions, including singing-based ones, might not only increase GM volume but also prevent further brain atrophy in PSA. However, future longitudinal studies with larger sample sizes are needed to elucidate whether the improved functional outcomes are underpinned by possible neuroprotective neuroplasticity changes that prevent GM atrophy and WM neurodegeneration poststroke, if not increase GM volumes and WM structural connectivity.

The observed relationship between the treatment-induced improvement of naming and neuroplasticity in the left frontal GM and WM is well in line with the classic models of word production (Indefrey and Levelt, 2004; Hickok, 2012) in which the speech motor processes of syllabification, phonetic encoding, and articulation are attributed to largely to these regions and pathways. The singing intervention may support this process by slowing the rate of word production and increasing the connectedness between syllables/words through continuous voicing and melodic intonation (Wan et al., 2010). Moreover, treatment-related modulation of the left-hemispheric cortical activity in Broca's area and the premotor cortex has been associated with improved naming in PSA (Fridriksson, 2010).

The present study has some potential limitations. First, the singing intervention comprised multiple components, and differentiating between the efficacies of individual treatment components is not possible based on the current data. Second, the sample size is relatively small and lacked an active control matched for dose of the intervention, limiting the generalizability of the findings. The sample size also affected the sensitivity of the additional voxel-wise analyses, where both focused analyses provided statistically significant results paralleling the a priori ROI-based analysis, but the unrestricted whole-brain analysis did not. Yet, the results from all the three voxel-wise analyses were similar and conformed with the ROI analysis. However, the beneficial effects of singing-based interventions might not be restricted to the left frontal regions, and future studies with larger sample sizes utilizing whole-brain analyses are warranted. Yet, this study is the largest multimodal RCT to date on treatment-induced neuroplasticity changes in PSA. While the present results need to be replicated in future larger studies, they are encouraging in providing us evidence of health economically promising multifaceted PSA treatment bringing about beneficial neuroplasticity change.

In conclusion, the present results suggest that the positive effects of singing on chronic PSA recovery are underpinned by structural GM and WM reorganization, mainly within the left frontal areas. Clinically, together with previous behavioral results on positive effects of singing in chronic PSA (Siponkoski et al., 2022), this evidence suggests that group-based singing is a feasible tool to promote language network reorganization and recovery in PSA.

## Synthesis

Reviewing Editor: Christoph Michel, Universite de Geneve Decisions are customarily a result of the Reviewing Editor and the peer reviewers coming together and discussing their recommendations until a consensus is reached. When revisions are invited, a fact-based synthesis statement explaining their decision and outlining what is needed to prepare a revision will be listed below. The following reviewer(s) agreed to reveal their identity: Massimo Filippi.

The two reviewers have a number of comments. They agree on several points, such as the necessity for a voxel wise analysis, the request to report all the data in a table, and the request to better define the clinical outcomes.

Both reviewers focused on suggesting improvement on the statistical analyses, results' presentation and interpretation.

The detailed comments of bothe reviewers are appended here:

Reviewer 1:

Summary

This manuscript investigates neural changes related to a group-based singing intervention in individuals with post-stroke aphasia. The authors report structural neuroimaging and behavioral language data from 28 individuals. They conclude that a singing intervention causes gray and white matter changes in left frontal areas.

While this manuscript presents an interesting and important area of research, I have several major concerns about the methods and results, as follows:

1. There is an under-reporting of data in this manuscript, which makes it difficult as a reviewer to evaluate the results and the authors' claims. The authors only present ANOVAs and subtractions of data between groups and timepoints. There are no data reported for any of the language or neural measures. WAB scores (Naming, Repetition) are reported for neither the control nor intervention group, and are not reported for either timepoint. In their analysis of putative WM tract changes, the authors do not report tract values in either group or at either of the timepoints and they do not report a p-value for this analysis. It is also not immediately clear from Figure 2 that there are any changes in WM tracts; it is not clear what the tracts visualized in this figure represent. Further, there is an absence of results reported for the GM analysis; GM volumes, with standard deviations, are not reported for either timepoint or group (again, only difference values are reported). An additional concern about the GM analysis is the decrease in GM volume in the control group. Is it possible this is just measurement error? The authors address this decrease in the Discussion and attribute it to brain atrophy following stroke. While it is certainly true that brain atrophy can occur after stroke, one wouldn't expect a group receiving standard rehabilitation care to show a decrease in GM volume, while a group receiving standard + singing therapy to show an increase in GM volume. In addition, the decrease in GM volume in the control group is seemingly driving the correlation visualized in Figure 3. Relatedly, several participants seem to show a decrease in WAB naming scores between T2 and T1 (this is assuming that 0 on the x-axis represents no change in score - it would be helpful if the authors could explain the axes in all figures). Why would patients show a decrease in WAB scores over time? Could this also just be measurement error/differences in test-retest performance?

2. A second main concern is with the degrees of analytical flexibility evidenced by the authors' analytical choices. As an example, the authors choose to include sex as a covariate in their partial correlation reported on line 313. This finding is tenuous (p=0.048) and it is unclear why the authors choose to include sex as a covariate in the model but not other demographic factors such as age, education, or time post-stroke? The omission of age is especially concerning because GM volumes are strongly modulated by age and this is a standard covariate in these kinds of analyses. This statistical choice - which provides the basis for one of the authors' main claims - is not well explained. Additionally, the rationale for choosing the WAB Naming and Repetition subtests as outcome measures is not well rationalized. The authors rationalize their use of these subtests based on "their established neuroanatomical grounds" (lines 149-150). This rationale does not seem appropriate. The rationale for using specific language measures should be based on what the intervention was aiming to target. What was the goal of the singing intervention? What aspect(s) of language was it meant to target, and how do the authors know that the intervention was successful in achieving this? The authors also justify the use of these subtests based on Siponkoski et al., 2022; however, this study seems to use the same dataset that is reported in the current manuscript (circular and confirmatory logic).

3. The authors do not provide a compelling neurobiological reason for why singing should cause gray and white matter changes in the first place. The authors mention that singing may be a neuroprotective factor; however, this is not further explained nor is it supported by the literature, to my understanding. It is not immediately clear to me, for example, why GM volume would decrease in a control group and increase in an intervention group (when both were receiving some types of therapy) and what the neural mechanism supporting that would be?

Other Comments:

• Please provide more details about the standard chronic stroke care (type of therapy, number of hours/frequency, etc.).

• How did the authors keep track of whether participants complied with the at-home singing program? I suggest that the authors include some (supplemental) individual-level data showing how often each participant practiced at home over the 16 weeks.

• It is not immediately clear to me how the 33 participants were selected from the group of 54, and at what stage in the process group randomization occurred. The authors note that 54 participants were enrolled and randomized in the trial (lines 100-101) but then say that 33 were recruited and then randomized (lines 114-115). Were all 54 participants randomized but then only 33 had usable MRI data? Was eligibility for the study determined after participants were randomized? It would be great if the authors could provide more clarity here.

• The authors should report how they determined their sample size. On line 273 they make a statement about this but it is not apparent where this is addressed in the manuscript.

• Table 1 has a section on music background. Can the authors include information about how this was assessed in the Methods?

• It is difficult to evaluate some of the methods in general. For example, it is not clear what exact type of model(s) were run and what variables were inputted based on this sentence in lines 247-251: "A nonparametric Spearman correlation model was built to derive the correlational tractography evaluating the longitudinal change of QA correlated with Group..."

• When the authors report "T1-T2" (line 281) and "ΔT2-T1" (line 288) are they referring to the same variable?

• Lines 278-281: What statistical test did the authors run? Was this one model or two models for the two WAB subtests?

• I suggest the authors conduct whole-brain analyses. There are regions important for music and language processing in the inferior parietal lobe, for example, that the authors neglect to investigate. In general, there are many other regions implicated in both singing and aphasia recovery beyond the four ROIs the authors focus on.

• I suggest citing Chen et al., 2023 who show that the language network does not support music perception. This stands in contrast to the literature the authors cite in lines 334-336.

Reviewer 2:

Post-stroke aphasia (PSA) is a common consequence both immediately after stroke and over time. In fact, 60% of patients exhibit PSA even one year after the neurological event, with a significant impact on the quality of life. This randomized control trial aimed to demonstrate the brain changes following singing-based treatment in chronic non-fluent aphasic patients. Twenty-eight patients were randomly assigned to the experimental or the control group. Both groups received the standard treatment for chronic PSA. Additionally to the standard care, the experimental group participated in weekly 90-minute group singing lessons for 16 weeks and engaged in home training three times a week for 30 minutes with the aid of a technological software.

Patients from both groups underwent a 3T MRI to evaluate structural grey and white matter neuroplasticity changes and their association with improvements in repetition and naming tasks of the Western Aphasia Battery (WAB). Four brain regions in each hemisphere were selected as ROIs based on their association with both singing intervention and aphasia recovery. Voxel-based morphometry demonstrated an increase in the volume of the left inferior frontal cortex in the singing group compared to the control group. Connectometry analysis revealed an enhancement of the structural white matter connectivity in the experimental group compared to the control group, specifically in the left arcuate fasciculus, corpus callosum and bilateral frontal aslant tract, superior longitudinal fasciculus and corticostriatal tract.

Furthermore, the experimental group demonstrated improved naming ability after the singing training and this improvement correlated with the increased volume of the left BA44, as well as with the arcuate fasciculus and the frontal aslant tract. In conclusion, these results suggest that group singing in PSA can lead to structural neuroplasticity changes in the left frontal language areas and bilateral language pathways, resulting in improved speech production even in a chronic phase.

This work is very interesting and contributes to the understanding of the neural underpinnings of improved language outcome, specifically after group-based singing treatment in chronic non-fluent aphasics. Nevertheless, I have some concerns:

1) Methods:

a) Among the inclusion criteria, authors included the presence of at least mild non-fluent aphasia. However, also in the abstract, it would be useful to specify that only patients with non-fluent aphasia were included. This clarification would allow a better understanding of the study and its target population.

b) From the manuscript, it appears that the authors only assessed naming and repetition abilities. However, if data have been acquired, it would be important to also report patients performances (and changes over time) on language comprehension, but also on extra-language domains, such as global cognitive functioning, attention, and memory.

c) For detecting gray matter changes, only four brain regions in each hemisphere were selected as ROIs based on their association with both singing intervention and aphasia recovery. Authors should also consider to perform a voxel-wise analysis in order to understand whether other changes in extra-language systems are present.

2) Results:

a) Authors mentioned that they focused their neuropsychological assessment on naming and repetition tasks. However, WAB scores were not reported in any table. Please insert a table containing all the WAB scores before and after the treatments to illustrate the absence of differences between groups at the baseline and the language improvements after the interventions. Furthermore, in the methods section, authors reported using the Aphasia Severity Rating Scale. It would be interesting to present the scores in a table to better define the baseline characteristic of the sample.

b) In Table 1, the Aphasia Quotient (AQ) is presented as an index to classify the severity of aphasia. However, it would be helpful to specify in the methods section how this score was defined.

Minor points:

a) Please, check the acronym PWA at lines 100 and 101 and the correct use of the English at lines 126 and 128.

b) In the legend of Table 1, please specify that group A is the experimental group and group B is the control group.

c) In Table 1, the BDAE Verbal Agility score is reported among the clinical characteristics of the sample but there is no reference to this score in the manuscript text.
