# Supplementary figures and images for: Structural Neuroplasticity Effects of Singing in Chronic Aphasia
Source: eNeuro. 2024 May 10;11(5):ENEURO.0408-23.2024. doi: 10.1523/ENEURO.0408-23.2024 (PMC11091951; doi:10.1523/ENEURO.0408-23.2024)

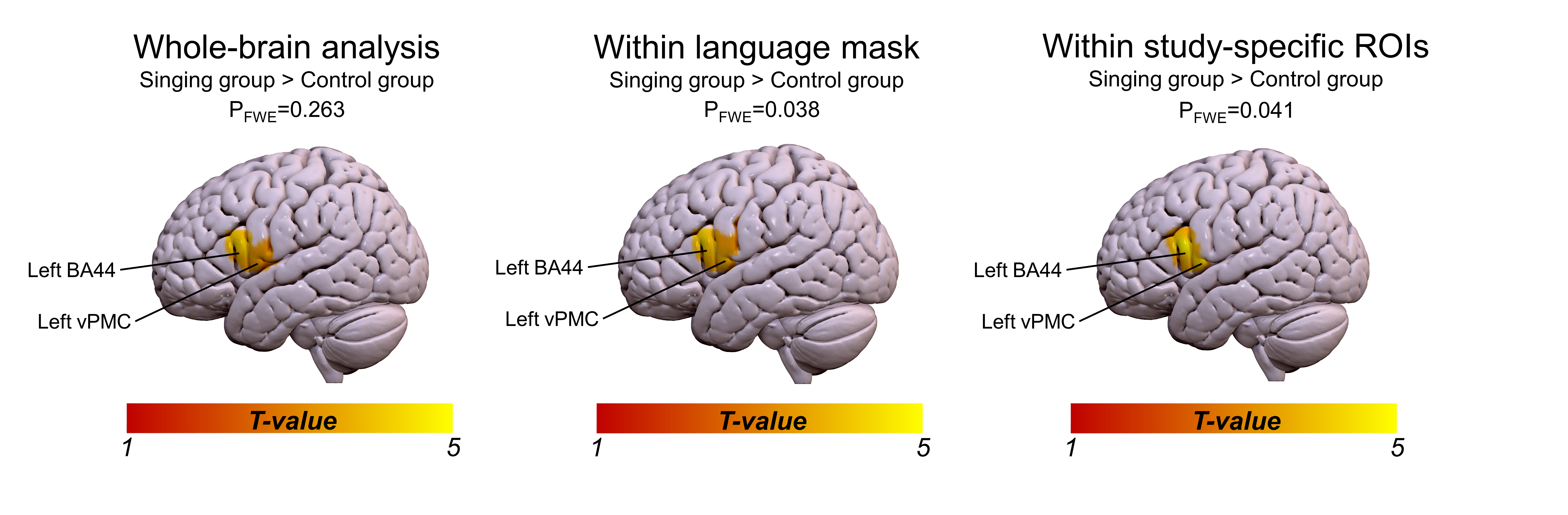

Supplement: Figure 4-1 — Voxel-wise analysis of the treatment-induced grey matter neuroplasticity changes. Longitudinal GM volume increases (Singing group>Control Group) in T2>T1 from (left) the whole-brain voxel-wise analysis, (middle) the voxel-wise analysis within the language network derived from meta-analysis (https://neurosynth.org/analyses/terms/language/) and (right) the voxel-wise analysis within the study-specific regions of interest. BA=Brodmann area, FWE=Family-wise error rate, vPMC=ventral premotor cortex. Download Figure 4-1, TIF file. [file eneuro-11-ENEURO.0408-23.2024-s002.tif]
